# Supplementary material for: Spray-Drying Microencapsulation of Bauhinia ungulata L. var. obtusifolia Aqueous Extract Containing Phenolic Compounds: A Comparative Study Using Different Wall Materials
Source: Pharmaceutics. 2024 Apr 2;16(4):488. doi: 10.3390/pharmaceutics16040488 (PMC11054010; doi:10.3390/pharmaceutics16040488)
Supplement: Supplementary file 1 [file pharmaceutics-16-00488-s001.zip › pharmaceutics-2828817-supplementary.pdf]

Supplementary Material

S1. Particle size distribution

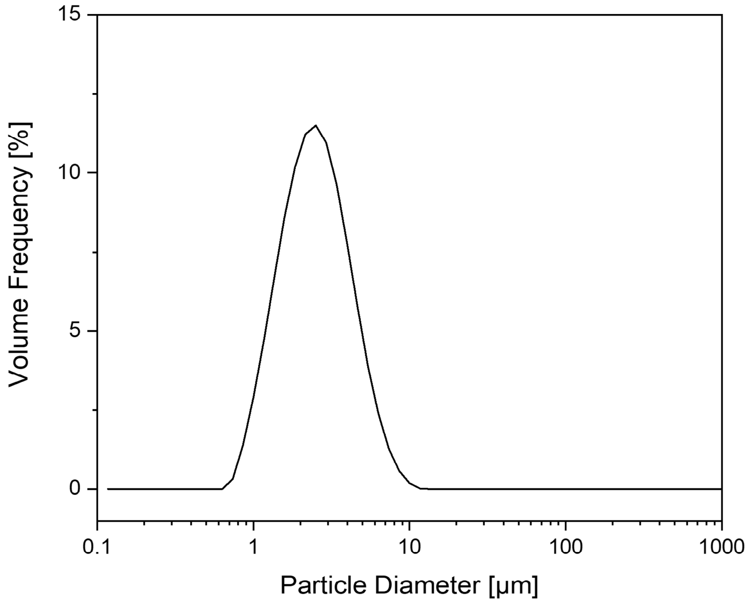

Figure S1. Particle size distribution of Bu-MD4.

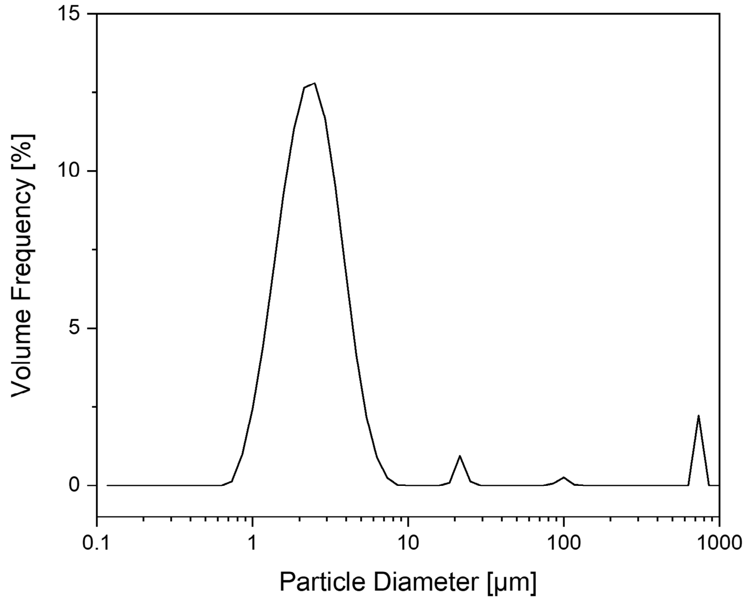

Figure S2. Particle size distribution of Bu-MD11.

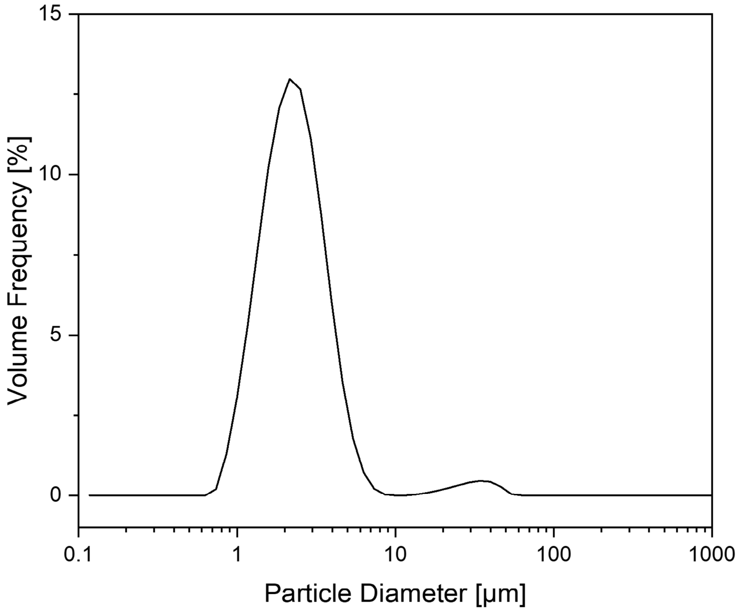

Figure S3. Particle size distribution of Bu-βCD.

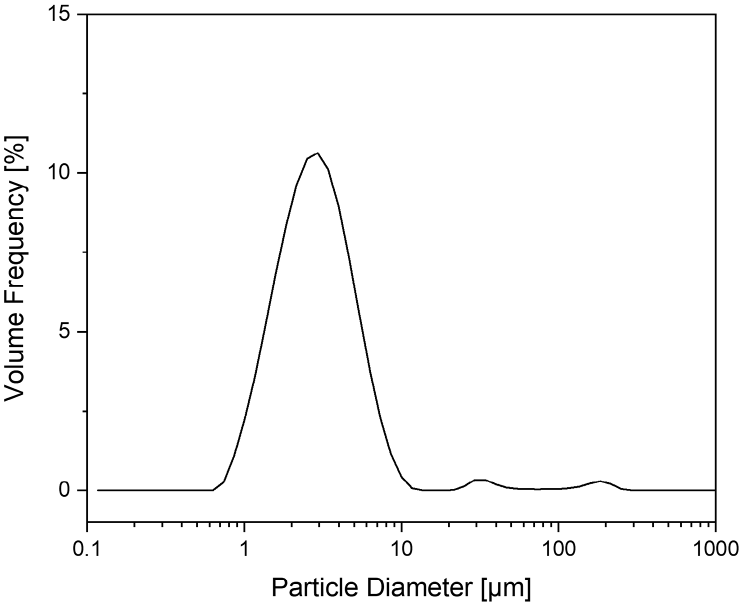

Figure S4. Particle size distribution of Bu-CMC.

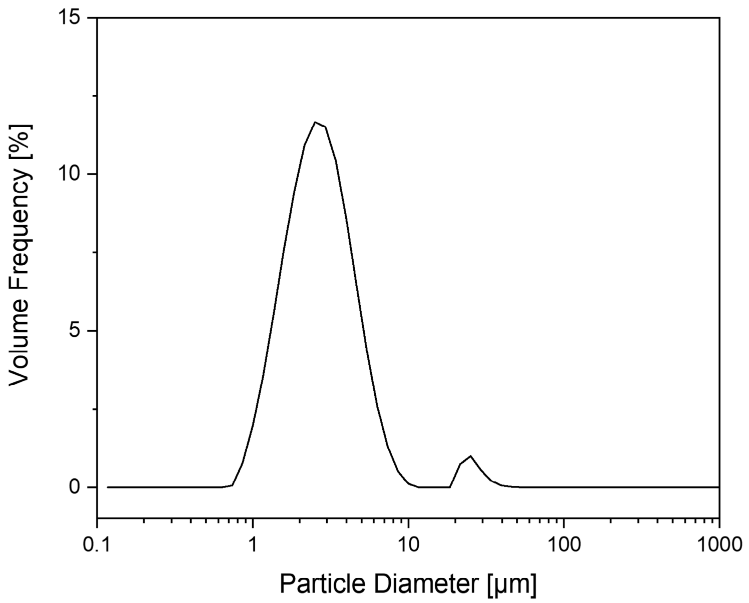

Figure S5. Particle size distribution of Bu-Pec.

S2. Differential scanning calorimetry

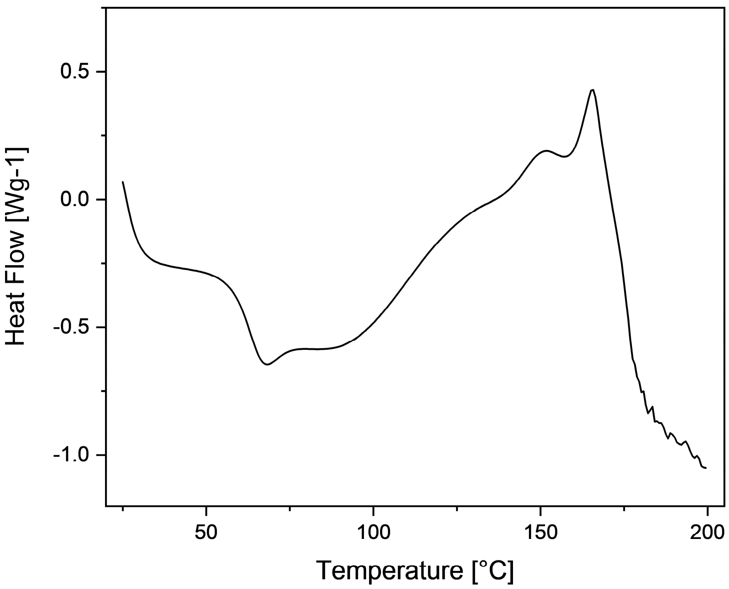

Figure S6. DSC thermogram of Bu-A (^exo).

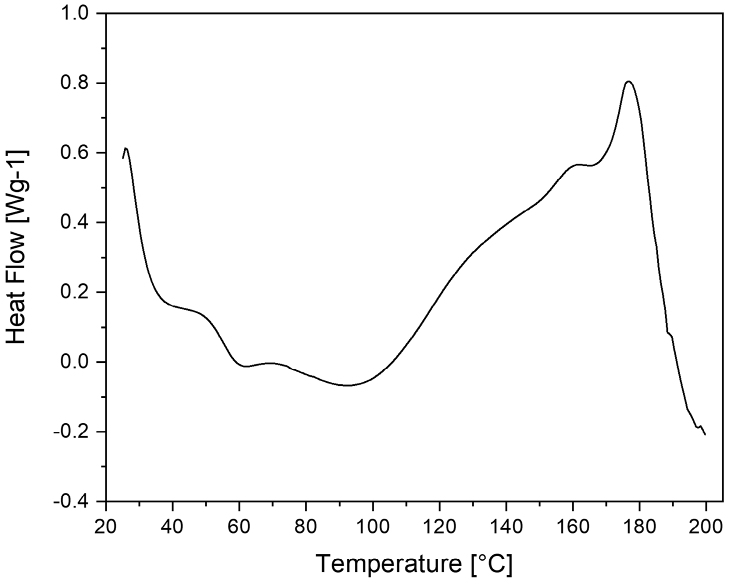

Figure S7. DSC thermogram of Bu-MD4 (^exo).

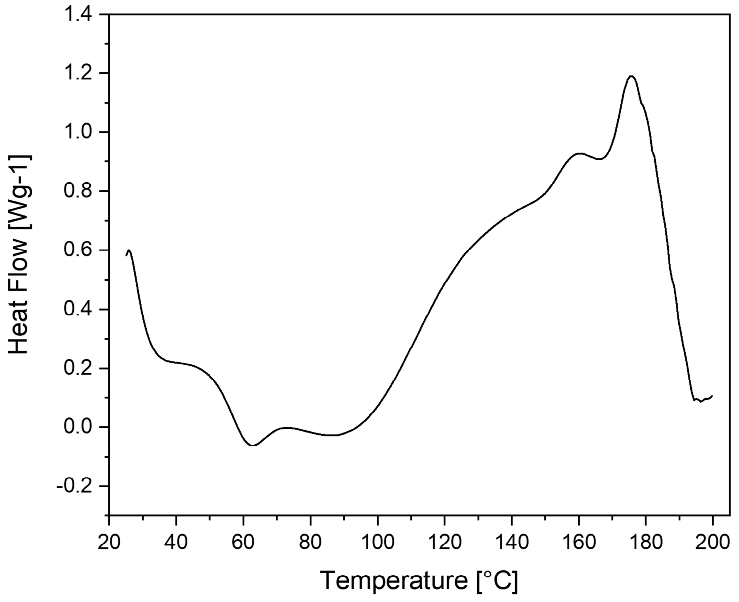

Figure S8. DSC thermogram of Bu-MD11 (^exo).

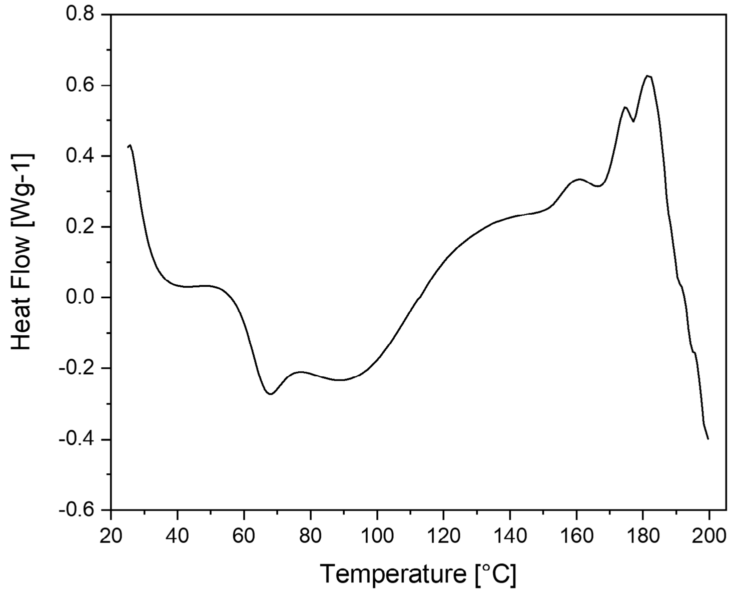

Figure S9. DSC thermogram of Bu-βCD (^exo).

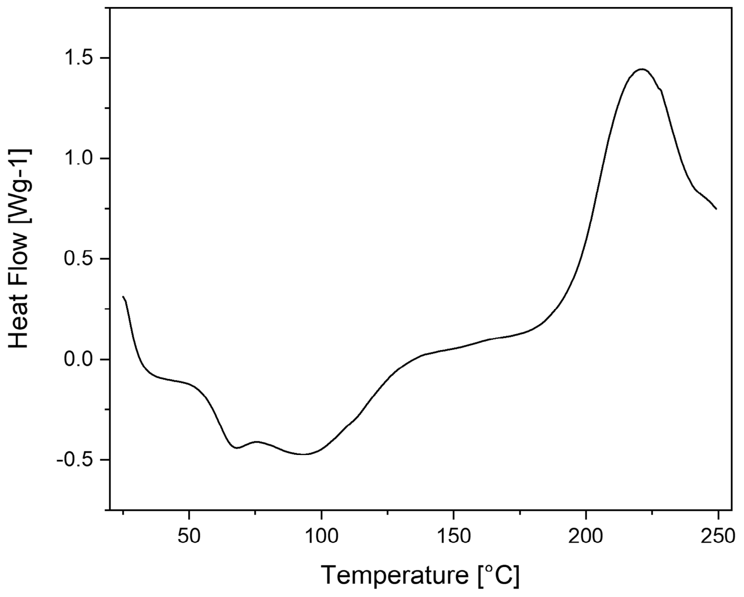

Figure S10. DSC thermogram of Bu-CMC (^exo).

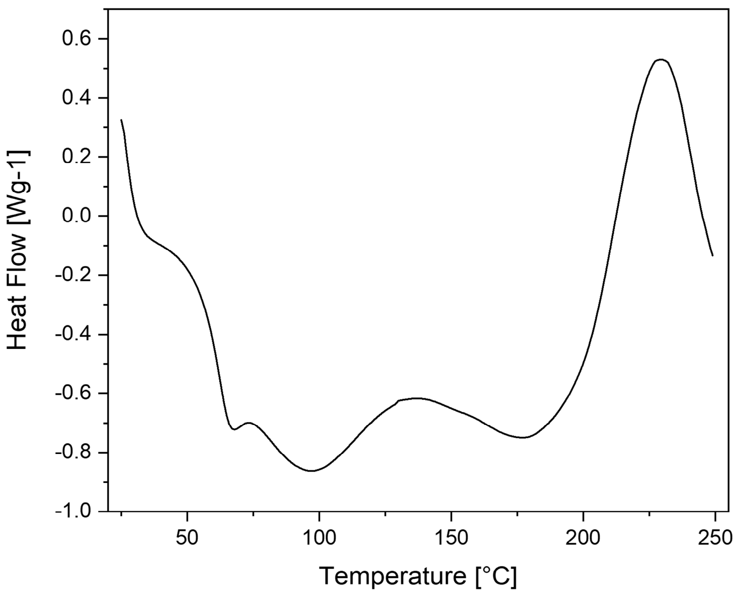

Figure S11. DSC thermogram of Bu-Pec (^exo).

S3. Statistical analysis

Table S1. Tukey's post-hoc comparison between the viscosity of the solutions to be sprayed without (Bu-A) or with wall material (Bu-MD4, Bu-MD11, Bu-βCD, Bu-Pec and Bu-CMC).

Comparison	p-value
Bu-A vs. Bu-CMC	<0.0001
Bu-MD4 vs. Bu-CMC	<0.0001
Bu-11 vs. Bu-CMC	<0.0001
Bu-βCD vs. Bu-CMC	<0.0001
Bu-CMC vs. Bu-Pec	<0.0001

Table S2. Tukey's post-hoc comparison between the particle size distribution of the SD microparticles containing the wall material.

Parameter	Comparison	p-value
Dv_{50}	Bu-βCD vs. Bu-CMC	0.0242
Dv_{90}	Bu-βCD vs. Bu-CMC	0.0457

Table S3. Tukey's post-hoc comparison between the moisture content of the SD microparticles.

Comparison	p-value
Bu-A vs. Bu-MD11	0.0310
Bu-A vs. Bu-CMC	0.0029
Bu-A vs. Bu-Pec	0.0104

Table S4. Tukey's post-hoc comparison between the initial decomposition temperature of the SD microparticles.

Comparison	p-value
Bu-A vs. Bu-CMC	<0.0001
Bu-A vs. Bu-Pec	<0.0001
Bu-MD4 vs. Bu-CMC	<0.0001
Bu-MD4 vs. Bu-Pec	<0.0001
Bu-MD11 vs. Bu-CMC	<0.0001
Bu-MD11 vs. Bu-Pec	<0.0001
Bu-βCD vs. Bu-CMC	<0.0001
Bu-βCD vs. Bu-Pec	<0.0001

Table S5. Tukey's post-hoc comparison between the total flavonoid content in the extract Bu-L and the SD microparticles.

Comparison	p-value
Bu-L vs. Bu-MD4	<0.0001
Bu-L vs. Bu-MD11	<0.0001
Bu-L vs. Bu-βCD	<0.0001
Bu-L vs. Bu-CMC	0.0455
Bu-A vs. Bu-MD4	<0.0001
Bu-A vs. Bu-MD11	<0.0001
Bu-A vs. Bu-βCD	<0.0001
Bu-MD4 vs. Bu-CMC	<0.0001
Bu-MD4 vs. Bu-Pec	<0.0001
Bu-MD11 vs. Bu-CMC	<0.0001
Bu-MD11 vs. Bu-Pec	<0.0001
Bu-βCD vs. Bu-CMC	<0.0001
Bu-βCD vs. Bu-Pec	<0.0001
Bu-CMC vs. Bu-Pec	0.0149

Table S6. Tukey's post-hoc comparison between the total phenolic content on the lyophilized extract (Bu-L) and the SD microparticles.

Comparison	p-value
Bu-L vs. Bu-MD4	0.0001
Bu-L vs. Bu-MD11	0.0108
Bu-L vs. Bu-βCD	0.0002
Bu-L vs. Bu-CMC	<0.0001
Bu-L vs. Bu-Pec	<0.0001
Bu-A vs. Bu-MD4	0.0001
Bu-A vs. Bu-MD11	0.0120
Bu-A vs. Bu-βCD	0.0002
Bu-A vs. Bu-CMC	<0.0001
Bu-A vs. Bu-Pec	<0.0001
Bu-MD4 vs. Bu-CMC	0.0275
Bu-MD4 vs. Bu-Pec	0.0008
Bu-MD11 vs. Bu-CMC	0.0003
Bu-MD11 vs. Bu-Pec	<0.0001
Bu-βCD vs. Bu-CMC	0.0191
Bu-βCD vs. Bu-Pec	0.0006

Table S7. Tukey's post-hoc comparison between the total phenolic content on the microparticles surface (PS) and encapsulation yield (YE) of the SD microparticles.

Parameter	Comparison	p-value
PS	Bu-MD4 vs. Bu-CMC	0.0016
YE	Bu-MD4 vs. Bu-CMC	0.0485
	Bu-MD4 vs. Bu-Pec	0.0035
	Bu-MD11 vs. Bu-CMC	0.0016
	Bu-MD11 vs. Bu-Pec	0.0002
	Bu-βCD vs. Bu-CMC	0.0369
	Bu-βCD vs. Bu-Pec	0.0027

Table S8. Tukey's post-hoc comparison between the encapsulation efficiency of the SD microparticles.

Comparison	p-value
Bu-MD4 vs. Bu-MD11	0.0072
Bu-MD4 vs. Bu-Pec	0.0427
Bu-MD11 vs. Bu-CMC	0.0154
Bu-MD11 vs. Bu-Pec	<0.0001
Bu-βCD vs. Bu-Pec	0.0020
Bu-CMC vs. Bu-Pec	0.0195

Table S9. Tukey's post-hoc comparison between the scavenging percentages and TAA against DPPH˙ radicals.

Scavenging percentages		TAA	
**Comparison**	**p-value**	**Comparison**	**p-value**
Bu-L vs. Bu-A	0.0068	Bu-L vs. Bu-MD4	<0.0001
Bu-L vs. Bu-MD4	<0.0001	Bu-L vs. Bu-MD11	<0.0001
Bu-L vs. Bu-MD11	<0.0001	Bu-L vs. Bu-βCD	<0.0001
Bu-L vs. Bu-βCD	<0.0001	Bu-L vs. Bu-CMC	<0.0001
Bu-L vs. Bu-CMC	<0.0001	Bu-L vs. Bu-Pec	<0.0001
Bu-L vs. Bu-Pec	<0.0001	Bu-A vs. Bu-MD4	0.0035
Bu-A vs. Bu-MD4	0.0005	Bu-A vs. Bu-MD11	<0.0001
Bu-A vs. Bu-MD11	<0.0001	Bu-A vs. Bu-βCD	<0.0001
Bu-A vs. Bu-βCD	<0.0001	Bu-A vs. Bu-CMC	<0.0001
Bu-A vs. Bu-CMC	<0.0001	Bu-A vs. Bu-Pec	<0.0001
Bu-A vs. Bu-Pec	<0.0001	Bu-MD4 vs. Bu-MD11	<0.0001
Bu-MD4 vs. Bu-MD11	<0.0001	Bu-MD4 vs. Bu-βCD	<0.0001
Bu-MD4 vs. Bu-βCD	<0.0001	Bu-MD4 vs. Bu-CMC	0.0001
Bu-MD4 vs. Bu-CMC	<0.0001	Bu-MD4 vs. Bu-Pec	0.0033
Bu-MD4 vs. Bu-Pec	0.0016	Bu-MD11 vs. Bu-Pec	0.0035
Bu-MD11 vs. Bu-Pec	0.0047	Bu-βCD vs. Bu-Pec	0.0047
Bu-βCD vs. Bu-Pec	0.0062	-	-

Table S10. Tukey's post-hoc comparison between the scavenging percentages and TAA against $ABTS^{\cdot+}$ radicals.

**Scavenging percentages**		**TAA**	
**Comparison**	**p-value**	**Comparison**	**p-value**
Bu-L vs. Bu-A	0.0005	Bu-L vs. Bu-A	0.0004
Bu-L vs. Bu-CMC	<0.0001	Bu-L vs. Bu-CMC	<0.0001
Bu-L vs. Bu-Pec	<0.0001	Bu-L vs. Bu-Pec	<0.0001
Bu-A vs. Bu-MD4	0.0004	Bu-A vs. Bu-MD4	0.0003
Bu-A vs. Bu-MD11	0.0052	Bu-A vs. Bu-MD11	0.0040
Bu-A vs. Bu-βCD	0.0014	Bu-A vs. Bu-βCD	0.0012
Bu-A vs. Bu-CMC	0.0221	Bu-A vs. Bu-CMC	0.0073
Bu-MD4 vs. Bu-CMC	<0.0001	Bu-MD4 vs. Bu-CMC	<0.0001
Bu-MD4 vs. Bu-Pec	<0.0001	Bu-MD4 vs. Bu-Pec	<0.0001
Bu-MD11 vs. Bu-CMC	<0.0001	Bu-MD11 vs. Bu-CMC	<0.0001
Bu-MD11 vs. Bu-Pec	0.0001	Bu-MD11 vs. Bu-Pec	<0.0001
Bu-βCD vs. Bu-CMC	<0.0001	Bu-βCD vs. Bu-CMC	<0.0001
Bu-βCD vs. Bu-Pec	<0.0001	Bu-βCD vs. Bu-Pec	<0.0001

Table S11. Tukey's post-hoc comparison between the content on chlorogenic acid in the lyophilized extract (Bu-L) and the SD microparticles.

µg/mL		mg/g	
**Comparison**	**p-value**	**Comparison**	**p-value**
Bu-L vs. Bu-A	<0.0001	Bu-L vs. Bu-A	<0.0001
Bu-L vs. Bu-MD4	<0.0001	Bu-L vs. Bu-MD4	0.0155
Bu-L vs. Bu-MD11	0.0008	Bu-L vs. Bu-βCD	0.0009
Bu-L vs. Bu-βCD	<0.0001	Bu-L vs. Bu-CMC	<0.0001
Bu-L vs. Bu-CMC	<0.0001	Bu-L vs. Bu-Pec	<0.0001
Bu-L vs. Bu-Pec	<0.0001	Bu-A vs. Bu-MD4	<0.0001
Bu-A vs. Bu-MD4	<0.0001	Bu-A vs. Bu-MD11	<0.0001
Bu-A vs. Bu-MD11	<0.0001	Bu-A vs. Bu-βCD	0.0001
Bu-A vs. Bu-βCD	<0.0001	Bu-A vs. Bu-CMC	0.0044
Bu-A vs. Bu-CMC	0.0007	Bu-A vs. Bu-Pec	0.0086
Bu-A vs. Bu-Pec	0.0014	Bu-MD4 vs. Bu-CMC	0.0339
Bu-MD4 vs. Bu-Pec	0.0444	Bu-MD4 vs. Bu-Pec	0.0176
Bu-MD11 vs. Bu-CMC	0.0063	Bu-MD11 vs. Bu-CMC	0.0039
Bu-MD11 vs. Bu-Pec	0.0028	Bu-MD11 vs. Bu-Pec	0.0020

Table S12. Tukey's post-hoc comparison between the content on rutin in the lyophilized extract (Bu-L) and the SD microparticles.

µg/mL		mg/g	
**Comparison**	**p-value**	**Comparison**	**p-value**
Bu-L vs. Bu-A	<0.0001	Bu-L vs. Bu-A	<0.0001
Bu-L vs. Bu-MD4	0.0014	Bu-L vs. Bu-βCD	0.0018
Bu-L vs. Bu-MD11	<0.0001	Bu-L vs. Bu-CMC	0.0011
Bu-L vs. Bu-βCD	<0.0001	Bu-L vs. Bu-Pec	<0.0001
Bu-L vs. Bu-CMC	<0.0001	Bu-A vs. Bu-MD4	<0.0001
Bu-L vs. Bu-Pec	<0.0001	Bu-A vs. Bu-MD11	<0.0001
Bu-A vs. Bu-MD4	<0.0001	Bu-A vs. Bu-βCD	<0.0001
Bu-A vs. Bu-MD11	<0.0001	Bu-A vs. Bu-CMC	<0.0001
Bu-A vs. Bu-βCD	<0.0001	Bu-A vs. Bu-Pec	<0.0001
Bu-A vs. Bu-CMC	<0.0001	Bu-MD4 vs. Bu-βCD	0.0136
Bu-A vs. Bu-Pec	<0.0001	Bu-MD4 vs. Bu-CMC	0.0086
Bu-MD4 vs. Bu-βCD	0.0139	Bu-MD4 vs. Bu-Pec	0.0003
Bu-MD4 vs. Bu-CMC	0.0298	Bu-MD11 vs. Bu-Pec	0.0087
Bu-MD4 vs. Bu-Pec	0.0006	-	-
Bu-MD11 vs. Bu-Pec	0.0113	-	-
